# Preventing misdiagnosis of diabetes in the elderly: age-dependent HbA1c reference intervals derived from two population-based study cohorts

**DOI:** 10.1186/s12902-019-0338-7

**Published:** 2019-02-12

**Authors:** Annette Masuch, Nele Friedrich, Johannes Roth, Matthias Nauck, Ulrich Alfons Müller, Astrid Petersmann

**Affiliations:** 1 Institute of Clinical Chemistry and Laboratory Medicine, University Medicine Greifswald, Ferdinand-Sauerbruch-Straße, 17475 Greifswald, Germany; 2 German Center for Cardiovascular Research (DZHK e.V.), partner site Greifswald, Greifswald, Germany; 3 Department Internal Medicine III, Endocrinology and Metabolic Diseases, University Hospital Jena, Jena, Germany; 4 Present address: Department of Anesthesiology and Intensive Care Medicine, Jena University Hospital, Jena, Germany

**Keywords:** HbA1c, Upper reference limit, Elderly, Diabetes diagnosis, Age-dependency

## Abstract

**Background:**

Measurement of gylcated hemoglobin A1c (HbA1c) plays a central role in monitoring quality of antidiabetic therapy and in the diagnosis of diabetes. Several studies report increased levels of HbA1c in nondiabetic elderly. However, this observation did not reach incorporation into daily clinical practice or the respective guidelines. The present study aimed to evaluate HbA1c levels in relation to age in two independent population-based cohorts and to derive age-specific reference intervals.

**Methods:**

Four thousand two hundred sixty three participants from the Study of Health in Pomerania (SHIP-0) and 4402 participants from the independent study SHIP-Trend were included. HbA1c was determined by means of high-performance liquid chromatography. Multivariable linear regression models were performed. Reference intervals for HbA1c were determined.

**Results:**

Reference intervals were derived from a healthy subpopulation with the upper reference limit (URL) for HbA1c of 42.1 mmol/Mol (6.0%) for individuals aged 20–39 years increasing to 43.2 mmol/Mol (6.1%) for individuals aged 40–59 years. For people aged ≥60 years the URL was 47.5 mmol/Mol (6.5%). In both study populations an increase in HbA1c with age was observed. ANOVA revealed up to 8.5 mmol/Mol (0.77%) or 7.3 mmol/Mol (0.68%) higher estimated mean levels of HbA1c in the oldest compared to the youngest age group in SHIP-0 or SHIP-trend, respectively. Linear regression analyses confirmed the positive associations of HbA1c with age which was independent of BMI

**Conclusion:**

The present study confirmed the previously observed increase of HbA1c with increasing age in non-diabetic individuals. As a consequence age-dependent reference values for HbA1c were derived from two large and well defined reference populations. Implementation of them into daily practice may improve patient care and diagnosis of diabetes and reduce the risk of misdiagnosis and subsequent overtreatment of diabetes in elderly patients.

## Background

Determination of glycated hemoglobin (HbA1c) is given a central role in the monitoring of antihyperglycemic therapy. In daily practice, the advantages of HbA1c include less day to day variability during acute illness and greater convenience as fasting is not required compared to fasting plasma glucose measurements and oral glucose tolerance tests. During the last decade, guidelines implemented HbA1c as equal diagnostic criterion besides measurement of plasma glucose for the diagnosis of diabetes as long as the HbA1c method is certified by the National Glycohemoglobin Standardization Program (NGSP) and is traceable to the Diabetes Control and Complications Trial (DCCT) reference assay.

The amount of HbA1c in the red blood cells (RBC) is directly related to the amount of plasma glucose as it is glycated in a non-enzymatic reaction [1]. However, measured HbA1c is also directly dependent on RBC life span, which may vary among individuals [2] and among different age groups [3]. RBC life span also appears to be reduced by hyperglycemia [4]. All RBC contribute to the measured level of HbA1c. Although older RBC are supposed to be exposed longer to blood glucose, younger RBC are more numerous [5]. Thus, HbA1c is considered a weighted measure of the average blood glucose levels during the past 120 days with plasma glucose levels from the preceding 30 days contributing substantially more (~ 50%) to the final result compared to plasma glucose levels from the past 90–120 days (~ 10%) [6]. Given the essential role of HbA1c in the diagnosis and management of diabetes it is paramount to understand physiological changes of HbA1c levels in relation to age in order to provide reasonable cut-offs as well as reference values. In this context is has to be noted that reference values for HbA1c were established in 1986 based on a small population of 124 nondiabetic individuals with a limited age range of 13–39 years [7]. These reference values have not been subject to change since then [8, 9]. The UKPDS found an HbA1c of 5.4% in 195 healthy persons 25–65 years and 5.6% in 53 healthy persons > 65 years, in contrast the manufacturers reference was given with 5.2 +/− 0.47% [10]. Although age-dependent differences in HbA1c were reported before, clinical guidelines currently in use still rely on reference values without accounting for this influence of age [11–13].

It is well known, that among the elderly, the prevalence of impaired glucose tolerance, impaired fasting glucose, and type 2 diabetes is increased [14]. Yet, glycemia and metabolic control change with age and several studies reported an increase of HbA1c in elderly non-diabetic individuals [15–22]. In line with this notion, also RBC lifespan appears to be affected by several aging-associated changes, e.g. alterations of the hematopoietic system [23], compromising either RBC production or clearance ultimately influencing HbA1c measures. However, clinicians so far found it difficult to incorporate this finding in daily practice not least because official guidelines do not stipulate HbA1c reference ranges or cut-offs for specific age groups.

Consequently, with respect to usage of a global cut-off for diagnosis of diabetes, disregarded age-related changes of HbA1c independent of disease might bear the risk of misdiagnosis in the elder population. Similarly, the HbA1c reference values for the monitoring of glycemia in patients with diabetes do not take the age of the individual into account potentially leading to unnecessary overtreatment with severe consequences [24, 25].

To improve the safety in application of HbA1c for the diagnosis of diabetes, non-diabetic individuals from two population-based cohorts were examined and compared with respect to age-specific changes in HbA1c levels providing HbA1c reference intervals for Caucasians in specific age-groups.

## Methods

### Study of health in Pomerania (SHIP)

SHIP (Study of Health in Pomerania) was designed to assess prevalence and incidence of common risk factors, subclinical disorders and clinical diseases; and, in addition, to investigate the complex associations among risk factors, subclinical disorders and clinical diseases. SHIP does not specifically address one selected disease but attempts to describe health-related conditions covering a wide focus to address the issue of overall less life-expectancy in this region in Germany [26]. The first cohort (SHIP-0) is based on representative samples of the population aged 20–79 years living in West Pomerania, a rural region in northeast Germany. The sampling was based on official data from population registries in the Federal State of Mecklenburg/West Pomerania. The baseline examinations in the SHIP cohort were performed between October 1997 and May 2001. Baseline examinations of the second independent cohort SHIP-Trend were conducted between September 2008 and September 2012. Participation in SHIP was exclusion criterion for SHIP-Trend. A second stratified (age, sex and city/county of residence) random sample of adults aged 20–79 years was drawn from population registries covering essentially the same area as SHIP-0 with only minor deviations. The rationale to perform a second study within the same region was to analyze the secular trend of subclinical and overt diseases and their determinants in a high-risk population and also to assess the prevalence of subclinical findings defined by highly innovative non-invasive methods (only within SHIP-Trend). The study design and sampling methods have been previously described in detail [26]. In the baseline examinations of the SHIP cohort, 4308 men and women from a representative population sample of 7008 subjects were examined. An additional 4420 men and women from a representative sample of 8016 adults participated in the baseline examinations of the independent SHIP-Trend cohort.

**Fig. 1:**
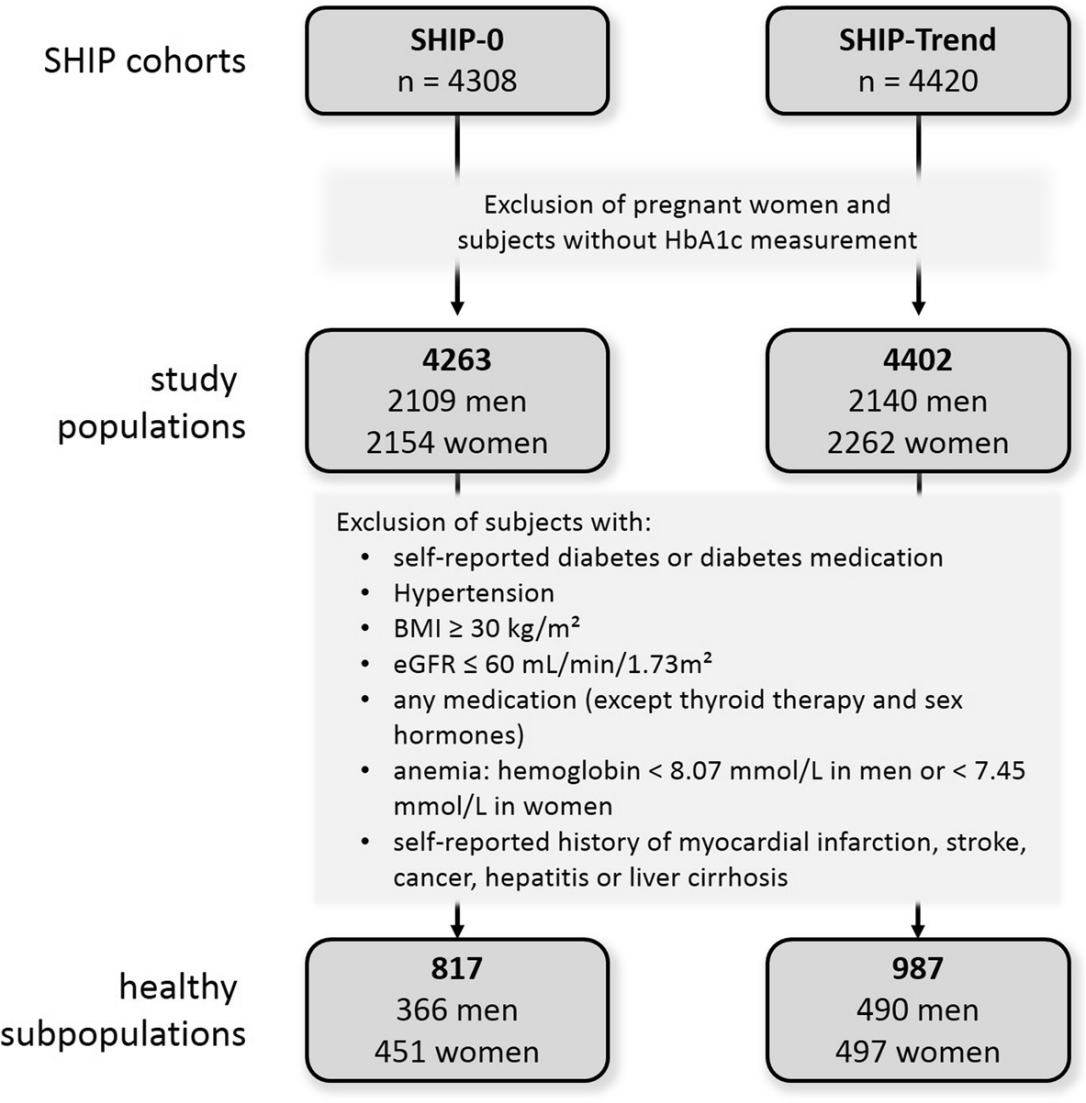
Flow diagram for the used independent study populations of SHIP-0 (Study of Health in Pomerania) and SHIP-Trend. All statistical models were applied to the total population as well as the healthy subpopulation. HbA1c reference values were derived from the combined healthy subpopulations

For the present analyses all pregnant women and subjects without HbA1c measurement were excluded resulting in 4263 SHIP-0 and 4402 SHIP-Trend participants. These populations formed the basis for our analyses. In a second step a healthy subpopulation was defined by excluding all subjects with at least one of the following condition (Fig. 1): self-reported diabetes mellitus or diabetes medication, hypertension, body mass index (BMI) ≥30 kg/m^2^, estimated glomerular filtration rate (eGFR) ≤60 mL/min/1.73m^2^, use of any medication identified by ATC code (except thyroid therapy [ATC H03] and sex hormones [ATC G03]), anemia based on low hemoglobin levels (men: < 8.07 mmol/L, women: < 7.45 mmol/L) as well as self-reported history of stroke, myocardial infarction, hepatitis, liver cirrhosis or cancer. The defined populations included 817 subjects in SHIP-0 and 987 subjects in SHIP-Trend. The healthy subpopulations were used for confirmatory reasons to exclude influences of obesity, selected medication or diseases as mentioned in Fig. 1 on the observed associations.

### Ethics, consent and permissions

All SHIP and SHIP-Trend participants gave written informed consent. Both studies follow the recommendations of the Declaration of Helsinki and were approved by the ethics committee of the University of Greifswald. Further, both studies were reviewed by an external scientific review board.

### Measurements

Subjects’ characteristics and medical histories including self-reported diabetes and medication were recorded using computer-aided personal interviews. Height and weight were measured without shoes and with light clothes. Body mass index (BMI) was calculated as weight (kg) divided by height (m) squared. After a 5-min rest period, systolic and diastolic blood pressure were measured three times in the right arm of seated subjects using a digital blood pressure monitor (HEM-705CP, Omron Corporation, Tokyo, Japan), with each reading followed by a rest period of 3 min. The last two readings were averaged to obtain the mean diastolic and systolic blood pressure. Hypertension was defined as a systolic blood pressure of ≥140 mmHg or a diastolic blood pressure of ≥90 mmHg.

Non-fasting (SHIP-0) or fasting (SHIP-Trend) blood samples were drawn from the cubital vein in the supine position and aliquots were prepared for immediate analysis and for storage at − 80 °C. HbA1c concentrations were determined by high-performance liquid chromatography (Bio-Rad Diamat, Munich, Germany). Serum cystatin C concentrations were measured using a nephelometric assay (Dimension VISTA, Siemens Healthcare Diagnostics, Eschborn, Germany) and the cystatin C-based eGFR was calculated using the CKD-EPI cystatin C equation [27]. All measurements complied with the regulations for internal and external quality controls according to the Guideline of the German Medical Association on Quality Assurance in Medical Laboratory Examinations (Rili-BAEK) [28].

### Statistical analysis

Continuous data are expressed as median (25th quartile, 75th quartile). Nominal data are expressed as percentage. Analysis of variance (ANOVA) was carried out to calculate adjusted means for HbA1c according to age groups. Multivariable linear regression models were performed to estimate independent associations of age as continuous variable with HbA1c. The models were adjusted for BMI. All models were performed in the whole study populations as well as in subpopulations of healthy subjects (see Fig. 1). Furthermore, the associations of age with HbA1c were assessed in BMI groups to rule out the role of BMI. To account for possible non-linear associations restricted cubic splines with three knots were used. The three knots were pre-specified located at the 5th, 50th, and 95th percentile as recommended by Stone and Koo [29] resulting in one component of the spline function: age^I^ in case of significant likelihood ratio test (*p* < 0.10). Statistical analyses were performed with SAS 9.4 (SAS Institute Inc., Cary, NC, USA).

## Results

### Population characteristics

General characteristics for the study populations are given in Table 1. SHIP-Trend participants were slightly older, less often current smoker and had higher BMI values compared to SHIP-0. Furthermore, SHIP-Trend subjects reported less often hypertension and had lower blood pressure. With respect to HbA1c, in both SHIP-0 [men: 5.4% (5.0, 5.9), 35.5 mmol/mol (31.1; 41.0); women: 5.3% (4.9, 5.7), 34.4 mmol/mol (30.1; 38.8); *p* < 0.01] and SHIP-Trend [men: 5.3% (5.0, 5.8), 34.4 mmol/mol (31.1; 39.1); women: 5.2% (4.8, 5.6), 33.3 mmol/mol (29.0; 37.7); *p* < 0.01] men showed higher HbA1c levels compared to women.

**Table 1:** General characteristics of the two independent study populations

	SHIP-0	SHIP-Trend	P*
Age (years)	50 (36; 63)	53 (40; 64)	< 0.01
Men (%)	49.5	48.6	0.42
Smoking (%)			< 0.01
Non-smoker	69.7	73.2	
Smoker	30.3	26.9	
Body-mass-index (kg/m^2^)	26.9 (23.8; 30.1)	27.5 (24.5; 31.0)	< 0.01
Self-reported Diabetes (%)	8.0	10.5	< 0.01
Self-reported diabetes medication (%)	8.0	8.8	0.21
Systolic blood pressure (mmHG)	135 (121; 149)	127 (115; 140)	< 0.01
Diastolic blood pressure (mmHG)	83 (76; 91)	77 (70; 84)	< 0.01
Hypertension (%)	45.0	26.7	< 0.01
eGFR (ml/min)	116 (102; 126)	111 (99; 121)	< 0.01

Data are expressed as median (25th percentile; 75th percentile) or percentages. *χ2-test (nominal data) or Mann-Whitney test (interval data) were performed. eGFR = estimated glomerular filtration rate

### Relation between age and HbA1c

**Fig. 2:**
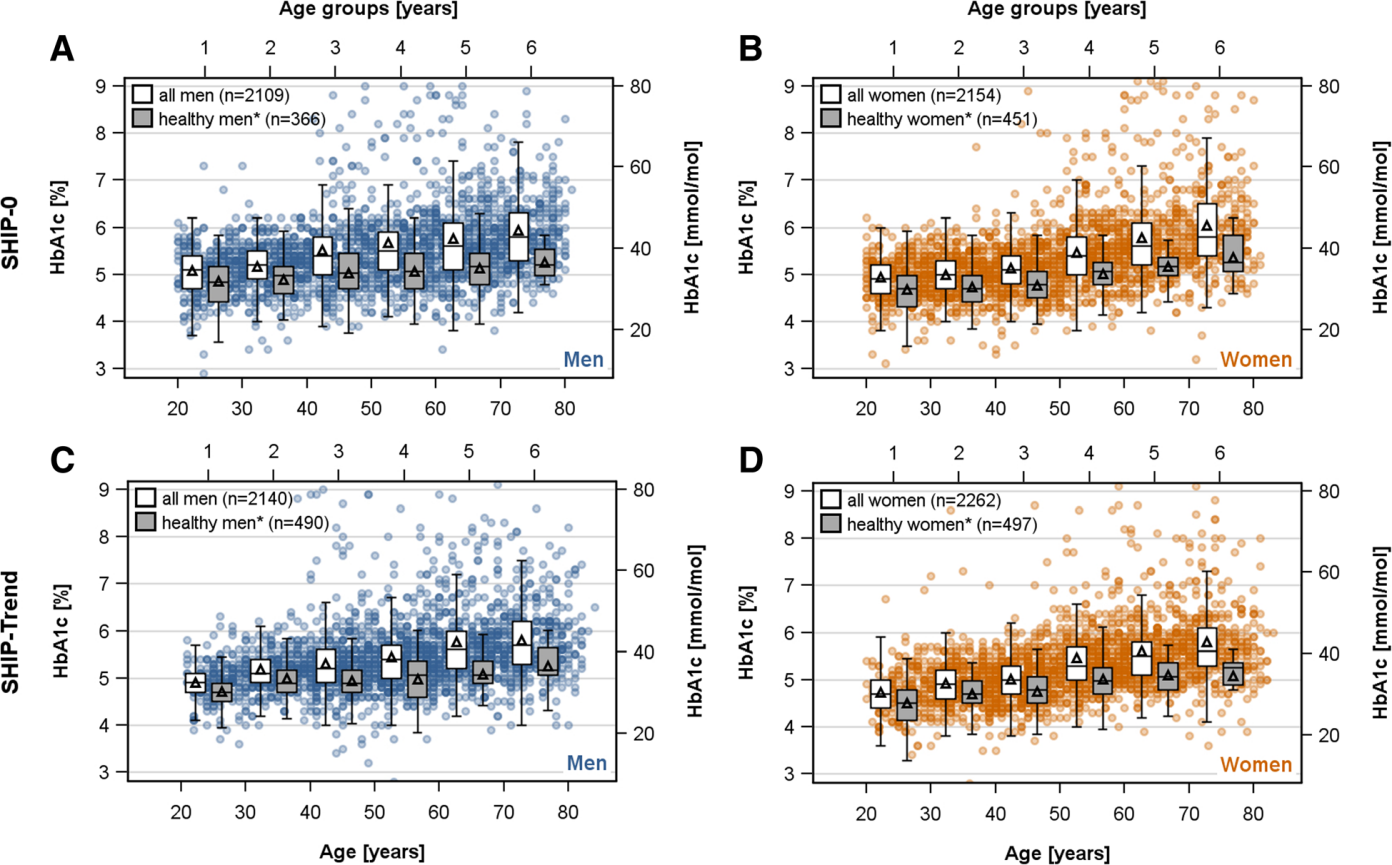
Sex-specific scatter plots of HbA1c against age as well as box plots of HbA1c by age groups in the SHIP-0 (**a**: men, **b**: women) and SHIP-Trend (**c**: men, **d**: women) population. The black triangle in the box plots indicates the mean value. The definition of healthy subjects (grey boxes) is given in Fig. 1

**Fig. 3:**
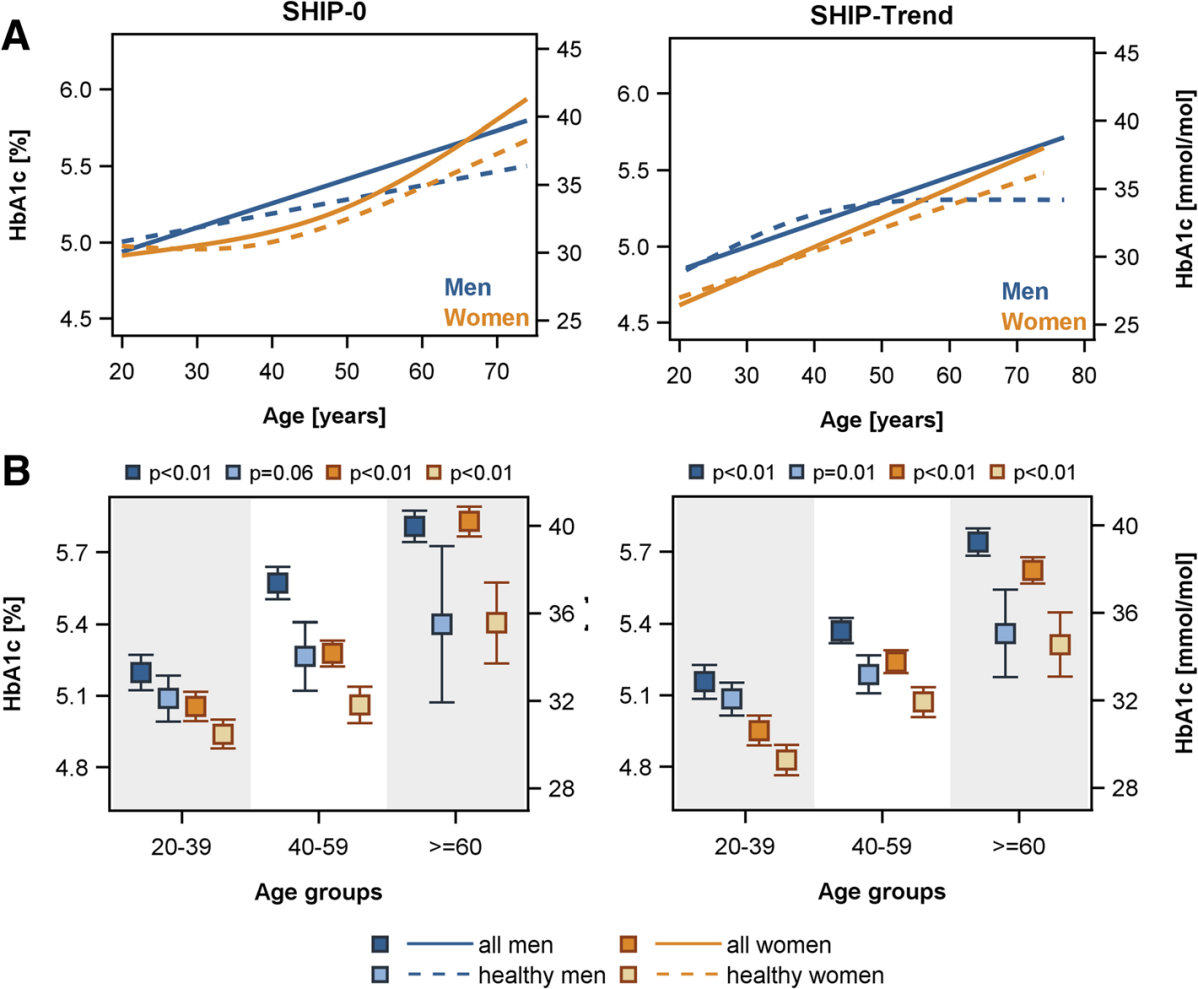
Sex-specific associations between age and HbA1c levels in the SHIP-0 and SHIP-Trend population. **a**) Linear regression analysis with restricted cubic splines (in case of significant likelihood ratio test, see method section) and **b**) estimated mean levels with 95% confidence interval assessed by analysis of variance (ANOVA). All models were adjusted for body mass index. The definition of healthy subjects is given in Fig. 1 and the regression estimates in Table 2

With respect to age, in both study populations an increase in HbA1c with age could be observed (Fig. 2) independent of the used study population (whole population [Fig. 2: white] or healthy subpopulation [Fig. 2: grey]). In detail, in the whole populations ANOVA revealed up to 0.78% or 0.67% higher estimated mean levels of HbA1c in the oldest [women: SHIP-0: 5.83% (95%-CI 5.77–5.89%); SHIP-Trend 5.62% (95%-CI 5.57–5.68%)] compared to the youngest [women: SHIP-0: 5.05% (95%-CI 4.99–5.12%); SHIP-Trend 4.95% (95%-CI 4.89–5.01%)] age group in SHIP-0 or SHIP-Trend, respectively (Fig. 3a). This corresponds to up to 8.5 mmol/mol or 7.3 mmol/mol higher estimated mean levels of HbA1c in the oldest [women: SHIP-0: 40.2 mmol/mol (95%-CI 39.5–40.9); SHIP-Trend 38.0 mol/mol (95%-CI 37.4–38.5)] compared to the youngest [women: SHIP-0: 31.7 mmol/mol (95%-CI 31.1–32.4); SHIP-Trend 30.6 mmol/mol (95%-CI 29.9–31.3)] age group in SHIP-0 or SHIP-Trend, respectively (Fig. 3a). Linear regression analyses confirmed the found positive associations of HbA1c with age (Table 2 and Fig. 3b). The exclusion of subjects with obesity, selected medication or diseases as described in Fig. 1 did not change the results (Table 2 and Fig. 3).

**Table 2:** Association between age and HbA1c [% (mmol/mol)] in two different populations

	SHIP-0			SHIP-Trend		
	beta	stderr	*p*	beta	stderr	*p*
Whole population						
Men						
age per 10 years	0.159 (1.74)	0.012 (0.14)	<.01	0.153 (1.67)	0.011 (0.12)	<.01
Women						
age per 10 years	0.094 (0.70)	0.030 (0.32)	0.03	0.191 (2.09)	0.011 (0.12)	<.01
age’	7.16E^−02^ (0.08)	1.40E^−02^ (0.02)	<.01	–	–	–
Healthy subpopulation*						
Men						
age per 10 years	0.094 (1.03)	0.032 (0.35)	<.01	0.211 (2.31)	0.057 (0.63)	<.01
age’	–	–	–	−0.011 (−0.12)	0.005 (0.06)	0.04
Women						
age per 10 years	−0.011 (− 0.12)	0.050 (0.55)	0.82	0.156 (1.70)	0.016 (0.18)	<.01
age’	8.63E^−02^ (0.09)	4.51E^−02^ (0.05)	0.06	–	–	–

Beta = beta coefficient, stderr = standard error. Linear regression models were adjusted for body mass index. Age’ represents spline components, for more detail see method section.*The definition of the healthy subpopulations is given in Fig. 1

**Fig. 4:**
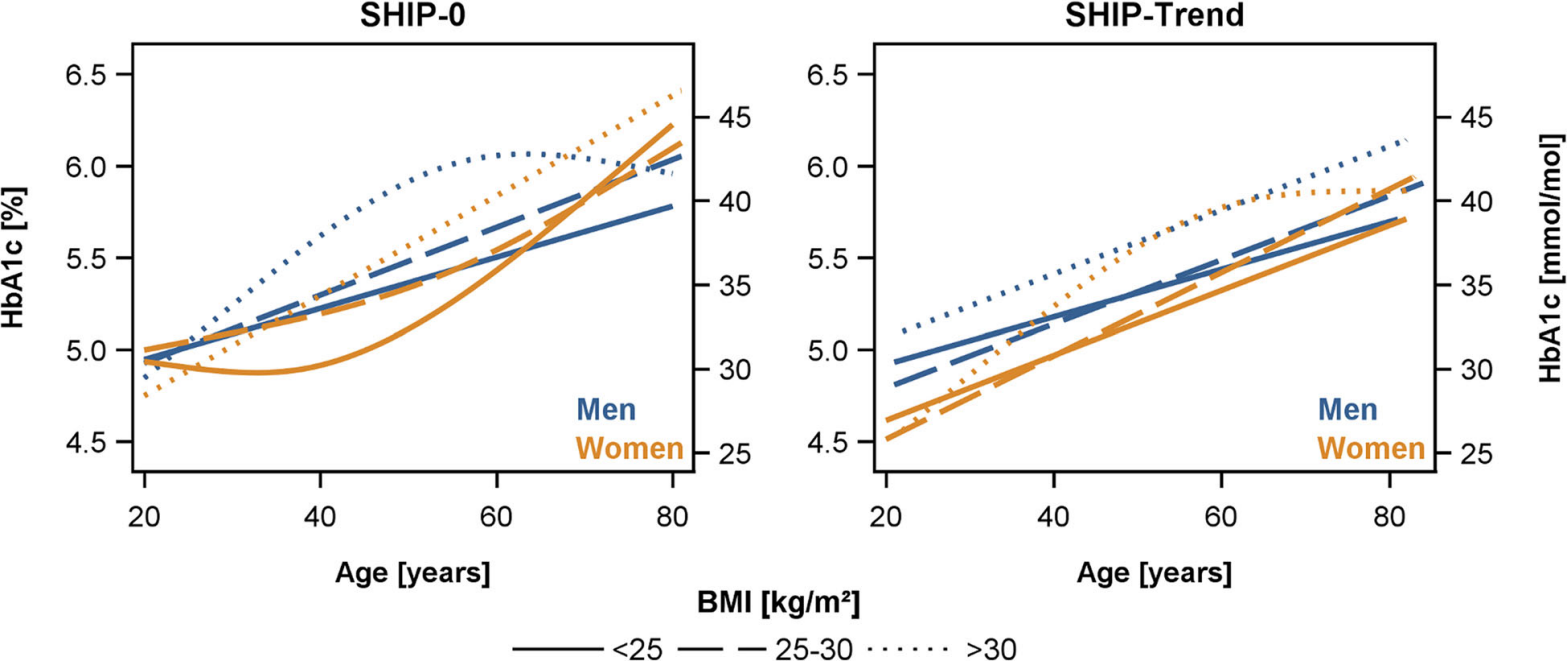
Sex-specific associations between age and HbA1c levels by groups of body mass index (BMI) in the whole SHIP-0 and SHIP-Trend population assessed by linear regression with restricted cubic splines (in case of significant likelihood ratio test, see Methods section)

Furthermore, the assessment of the association between age and HbA1c in subgroups according to the BMI (< 25, 25–30, > 30 kg/m^2^) revealed no differences in the direction and strength of the associations in lean, overweight and obese subjects (Fig. 4).

### Reference values for HbA1c in age-groups

Healthy subpopulations of SHIP-0 and SHIP-Trend were combined to derive reference intervals for HbA1c in different age-groups (Table 3). For individuals aged 20–39 years the upper reference limit (URL) for HbA1c was 6.0% (42.1 mmolmol) increasing to 6.1% (43.2 mmol/mol) for individuals aged 40–59 years while for people aged ≥60 years the URL was 6.5% (47.5 mmol/mol). Reference intervals for men and women differ only slightly. As mentioned above, women had lower HbA1c than men, in turn HbA1c URL for women are slightly lower than for men in each age group (Table 3).

**Table 3:** Reference intervals for HbA1c based on healthy population*

	2.5–97.5% percentile in mmol/mol (%)		
	All subjects	Men	Women
By age groups			
20–39 years	20.2–42.1 (4.0–6.0)	21.3–43.2 (4.1–6.1)	20.2–39.9 (4.0–5.8)
40–59 years	21.3–44.3 (4.1–6.2)	20.2–44.3 (4.0–6.2)	21.3–43.2 (4.1–6.1)
≥ 60 years	24.6–48.6 (4.4–6.6)	24.6–48.6 (4.4–6.6)	24.6–47.5 (4.4–6.5)

*The definition of the healthy subpopulations is given in Fig. 1. SHIP-0 and SHIP-Trend were combined for reference value calculation

## Discussion

The present study demonstrated the positive association of the HbA1c concentration with age in two independent population-based cohorts. This increase was observed in lean, overweight as well as obese individuals equally. Exclusion of subjects with obesity, medication intake or having other diseases as specified in Fig. 1 did not change this association substantially. Therefore, reference intervals were derived for specific age-groups on the basis of the healthy subpopulations of both cohorts.

The positive association of HbA1c with age was previously shown in several populations of different ethnicities [15, 17–20] and was confirmed in the Framingham Offspring study (FOS) and by analysis of the National Health and Nutrition Examination Survey (NHANES) 2001–2004 [21]. The early study of Arnetz et al. [15] was comparably small including only 48 subjects aging 50–89 years. Nevertheless, a significantly higher HbA1c concentration was found in older individuals compared to younger ones. Carrera and coworkers [19] examined a Mediterranean population including 1080 healthy individuals with HbA1c concentrations < 6.0% and report no differences between men and women in the whole population, but an overall increase of HbA1c over 10-years age-groups.

Our analysis showed an increase of HbA1c of 0.153% (1.7 mmol/mol) per decade in men and a comparable increase of 0.191% (2.1 mmol/mol) per decade in women in the SHIP-TREND. In the SHIP-0 cohort the rise per decade was comparable. Overall, these results are in line with previous data from non-diabetic patients recruited in an outpatient center showing a total increase of HbA1c from lowest (< 30 years) to highest (> 70 years) age-group of 0.47% [22], while in SHIP-0 an increase of 0.78% (8.5 mmol/mol, women) and in SHIP-TREND an increase of 0.67% (7.3 mmol/mol, women) was estimated. Also in the NHANES as well as the FOS samples comparable observations were reported [21]. For these samples upper 97.5th percentiles were determined for five-year age groups. In comparison to these data the reference values determined for the total population in the present study are very well in line with the upper 97.5th upper reference limits (URL) for the FOS samples from individuals with normal glucose tolerance [21] arguing for the generalizability of our data. Remarkably, for individuals aged ≥60 years the URL was 6.3% (45.4 mmol/mol) for men and 6.5% (47.5 mmol/mol) for women in the present study. Such high levels of HbA1c would currently lead to the diagnosis of diabetes according to the guidelines which do not take the increase of HbA1c with age into account [12, 13].

In a Japanese population of 7664 males aged 20–59 years the association between HbA1c and age was also dependent on BMI, especially in age-groups 30–39 years, but not on active participation in physical activity [17]. Our findings do not confirm this as no difference in strength and direction of the age-dependent increase in HbA1c across BMI groups (< 25, 25–30, > 30 kg/m^2^) was observed.

Notably, considerable variability of HbA1c exists independent of glycemia in non-diabetic populations [30, 31]. This phenomenon seems to be associated with the RBC life span. RBC with disease related reduced life span were reported to have lower HbA1c [32]. In line with this notion, Cohen et al. demonstrated that inter-individual variability in erythrocyte lifespan significantly influenced the HbA1c levels in diabetic as well as non-diabetic subjects [2]. These data have been further examined recently by Beltran del Rio et al. who proposed that RBC turnover is regulated by two main mechanisms: random cell loss and the senescence-mediated clearance of RBC from the circulation [33]. This contradicts the common notion that RBC exhibit only non-random removal from the circulation [34]. It may be conceivable that age-related changes in erythropoiesis, erythrocyte turn over or clearance contribute to variations in HbA1c with age independent of altered metabolic control. So far it is known that under steady-state conditions most elder people maintain a normal RBC count and normal erythropoiesis while under stress conditions the hematopoietic potential seems compromised in the aged [34]. Numerous conditions appear to influence RBC life span, e.g. aging-associated increased oxidative stress, which may enhance RBC removal from the circulation [34]. However, longitudinal studies examining RBC life span are missing. Senescent RBC are cleared from the circulation via phagocytosis by macrophages, likely Kupffer cells in the liver [35]. Notably, age-related impairment of the immune system including macrophage function has been recognized and is under intense research [36]. In this regard, impaired clearance of RBC due to compromised macrophage function and in turn prolonged exposure to blood glucose might contribute to the observed age-dependent increase in HbA1c levels independent of impaired metabolic control. Furthermore, iron deficiency and vitamin B12 deficiency are associated with increased HbA1c independent of hyperglycemia. Impaired splenic function is also known to increase HbA1c levels [5]. While the prevalence of iron as well as vitamin B12 deficiency rises with increasing age [37, 38], splenic function has been reported to decline with age [39]. Yet, at this point it is not possible to pin point specific reasons for the age-dependent increase in HbA1c levels and further studies are needed to clarify the underlying physiologic processes. Notably, genetic diseases like hemoglobinopathies and thalassemias may impact the interpretation of HbA1c measurement results as well. This needs to be considered especially in people from the African ancestry, the Mediterranean Basin, and from the Middle East and Southeast Asia in whom these inherited disorders have a higher prevalence. In this context, HbA1c measurement should be performed using an assay which is not affected by abnormal hemoglobin.

It is well described, that with increasing age even in adults without diabetes the hepatic, neurologic, endocrine, cardiac, and renal responses to hypoglycemia are compromised due to senescence impairing the counter-regulation systems. Of note, especially the autonomic system is muted which would have led to symptoms like hunger, diaphoresis, arousal, tremor, and palpitation via neurotransmitter release in response to hypoglycemia [40]. These ageing-related compromises are exacerbated in elderly with diabetes which might have severe consequences. In this regard, especially hypoglycemia due to inadequate HbA1c-targets in elderly demands attention. Consistent with this notion the retrospective study of Müller et al. analyzed data of the GUIDANCE study and identified potential overtreatment of elderly with diabetes, meaning intensive glycemic control and HbA1c targets at 6.5% independent of age or comorbidity [25]. In recent years, the recognized risk of overtreatment in elderly diabetic patients as wells as the increased risk of hypoglycemia led to the approach of personalized diabetes treatment rather than a generalized regime. This was included in the recommendation of the American Diabetes Association and the European Association for the Study of Diabetes in 2012 [41].

In line with this, our study provides age-dependent reference intervals for HbA1c for Caucasians. This may further improve patient care and safety of HbA1c assessment for the purpose of diabetes diagnosis. However, the established HbA1c cut-off for the diagnosis of diabetes was derived rather on the basis of the associated risk of microvascular and cardiovascular disease and not on the basis of population-based reference values. Thus, justification of age-dependent HbA1c diagnostic cut-offs needs further investigation with respect to the development of associated complications. Nevertheless, general awareness of age-related increases in HbA1c independent of diabetes may avoid overtreatment and misdiagnosis in the elder population. In addition, in regard of the numerous factors that may affect HbA1c levels independent of glycemia, it becomes increasingly clear that special care needs to be taken for HbA1c as a biomarker for the diagnosis of diabetes. Awareness needs to be raised for the variability that may occur among healthy individuals.

### Limitations

The populations of SHIP-0 and SHIP-Trend consist of Caucasians. The current consent in literature is that Caucasians have lowest HbA1c levels, Mexican American have higher HbA1c, while highest levels in HbA1c are observed in Blacks [1]. In turn, derived reference intervals for age-groups may be inappropriate for other ethnicities than Caucasians.

### Conclusions

In conclusion, the present study confirmed the previously observed increase of HbA1c with increasing age in non-diabetic individuals. This association between HbA1c and age was found to be independent of BMI. Underlying reasons remain to be elucidated. However, with reference values that disregard the age-related increase of HbA1c potential overtreatment and the risk of misdiagnosis of diabetes in elderly may be the consequence. Therefore, our study for the first time provides age-dependent reference values for HbA1c. Awareness of clinicians of the age-related increase of HbA1c independent of diabetes and the transition of this fact into age-dependent reference intervals may improve patient care and diagnosis of diabetes.

## Abbreviations

FOS: Framingham Offspring study
NHANES: National Health and Nutrition Examination Survey
RBC: red blood cell
SHIP: Study of Health in Pomerania
